# Integrating Client Tracker Tool Into Fistula Management: Experience From the Fistula Care Plus Project in the Democratic Republic of Congo, 2017 to 2019

**DOI:** 10.3389/fpubh.2022.902107

**Published:** 2022-06-09

**Authors:** Justin Paluku, Esther Kitambala, Cathy Mufungizi Furaha, Rita Bulu Bobina, Pascal Habamungu, Bienvenu Salim Camara, Sidikiba Sidibe, Don Félicien Banze Kyongolwa, Vandana Tripathi, Alexandre Delamou

**Affiliations:** ^1^Heal-Africa, Goma, Democratic Republic of Congo; ^2^Africa Center of Excellence for Communicable Diseases Prevention and Control (CEA-PCMT), University Gamal Abdel Nasser, Conakry, Guinea; ^3^Centre National de Formation et de Recherche en Santé Rurale de Maferinyah, Forécariah, Guinea; ^4^EngenderHealth, Kinshasa, Democratic Republic of Congo; ^5^EngenderHealth, New York, NY, United States

**Keywords:** Client Tracker, fistula management, Fistula Care Plus, Democratic Republic of Congo, implementation research

## Abstract

This study aimed to document the experience of integration and the contribution of the *Client Tracker* (CT) to female genital fistula (FGF) management and data quality in sites supported by the *Fistula Care*+ Project in the Democratic Republic of Congo (DRC), from 2017 to 2019. It was a parallel mixed methods study using routine quantitative data and qualitative data from in-depth interviews with the project staff. Quantitative findings indicated that CT forms were present in the medical records of 63% of patients; of these, 38% were completely filled out, and 29% were correctly filled out. Qualitative findings suggested that the level of use of CT in the management of FGF was associated with staff familiarity with the CT, staff understanding of concepts in the CT forms, and the CT-related additional workload. The CT has mainly contributed to improving data quality and reporting, quality of care, follow-up of fistula patients, and self-supervision of management activities. A possible simplification of the CT and/or harmonization of its content with existing routine forms, coupled with adequate continuous training of staff on record-keeping, would further contribute to maximizing CT effectiveness and sustainability.

## Introduction

The Client Tracker (CT) is a paper and electronic-based tool that clinicians use to collect information about the patient's care related to female genital fistula (FGF), pelvic organ prolapse, urinary incontinence, and other genital tract conditions (1). The CT is part of a The Surgical Safety Toolkit, which is a set of clinical monitoring tools and quality assurance checklists, designed to address data gaps in medical records and challenges for understanding clinical data trends (1). is The CT is therefore used to document client information and management practices to enable evaluation and scientific research activities to improve the clinical policies and practices of maternal health programs (1).

In the Democratic Republic of Congo (DRC), where the number of women living with untreated fistula was estimated at 42,000 in 2007 (2), EngenderHealth's USAID-funded Fistula Care *Plus* (FC+) Project in the Democratic Republic of Congo (DRC) introduced in 2017, the CT in the program to manage women with FGF (3). The CT was adapted to the country FGF management realities and translated into French by the DRC country office of EngenderHealth, then integrated in the DRC hospital information system. The introduction of the CT in FC+ sites aimed to improve FGF management in the DRC (3).

Indeed, various information-gathering tools have been integrated into the surgical management of patients to improve it. In 2009, the World Health Organization (WHO) developed and recommended using the surgical safety checklist for surgical problems management in developing countries (4). This checklist, used by health systems in many countries, reduces mistakes and adverse events and strengthens teamwork and communication in surgical care (4). In Guinea, an evaluation by the Robert Koch Institute of Berlin, Germany, showed that integrating the WHO's surgical safety checklist into patient management in the Surgical Department of the Faranah Regional Hospital helped improve teamwork and reduce patient management mistakes (5).

Improving FGF management is a priority in low- and middle-income countries (LMICs) where the high annual incidence of deliveries (124 cases per 100,000 deliveries in rural areas) contributes to maternal morbidity and leads to significant socioeconomic impacts on affected women and their families (6–8). Additionally, surgery—required for most FGF repair—has patient safety implication in LMICs (9). Integrating a CT tool to improve the quality of FGF management could improve data collection and management.

However, beyond project management expectations and the effects of the CT on FGF management and data quality at project-supported sites, it is important to investigate practicalities related to CT use to understand its effectiveness and sustainability. The use of information-gathering tools in surgical care has sometimes been limited by practical difficulties in different contexts (5). Therefore, it is useful to explore the CT-related workload and the need for additional staff skills for its optimal use.

The objective of this study was to document the experience of CT integration and its contribution to FGF management and data quality at FC+ sites in DRC from 2017 to 2019. Specifically, the study aimed to document the use, contribution, and sustainability of the CT for FGF management and data collection.

## Materials and Methods

### Study Design

This study was a parallel mixed methods study using quantitative and qualitative data. The quantitative data were used to assess the use of CT forms. In contrast, the qualitative data helped to understand the experience of integration and the CT's contribution to the management of FGF.

### Study Setting

#### General Setting

DRC is a Central African country covering 2,345,000 km^**2**^ with an estimated population of 84.1 million inhabitants in 2018, of which about 70% live in rural areas (10). The country has 515 health zones; the national health system is decentralized to the provincial level and financed through public and private mechanisms (11).

With an average of 6.1 children per woman, the country's total fertility rate is higher than the average for sub-Saharan Africa (11). The rate of early pregnancy is also high, with 125.24 births per 1,000 teenagers (15–19 years old) (10). The maternal mortality ratio in the DRC is estimated at 693 (CI of 509–1010) deaths per 100,000 live births, and the risk of death for a woman during her reproductive life is one in 24 women (12).

#### FGF Management at Fistula Care Plus Sites

FGFs management at FC+ project sites aligns with WHO's guidelines (13). As for the organization at each site, the FGF patient follows a particular pathway which includes reception by the counselors, clinical examination by doctors, para-clinical check-ups, anesthetic consultation, surgery, and postoperative care by nurses, physiotherapists, psychologists and counselors. The patient receives clinical visits every day until discharge. A counter-referral letter is issued if the patient has been transferred. Upon hospital discharge, the client is offered socio-economic reintegration and free family planning services.

Surgical management is provided either through routine (at FC+ sites) or outreach (at partner hospitals in almost every province of the country) services. Staff from FC+ sites travel to provide outreach services in other provinces. Records (including the CT) of patients managed in outreach settings are kept at the FC+ sites that provided the care.

#### Specific Settings

**Figure 1:**
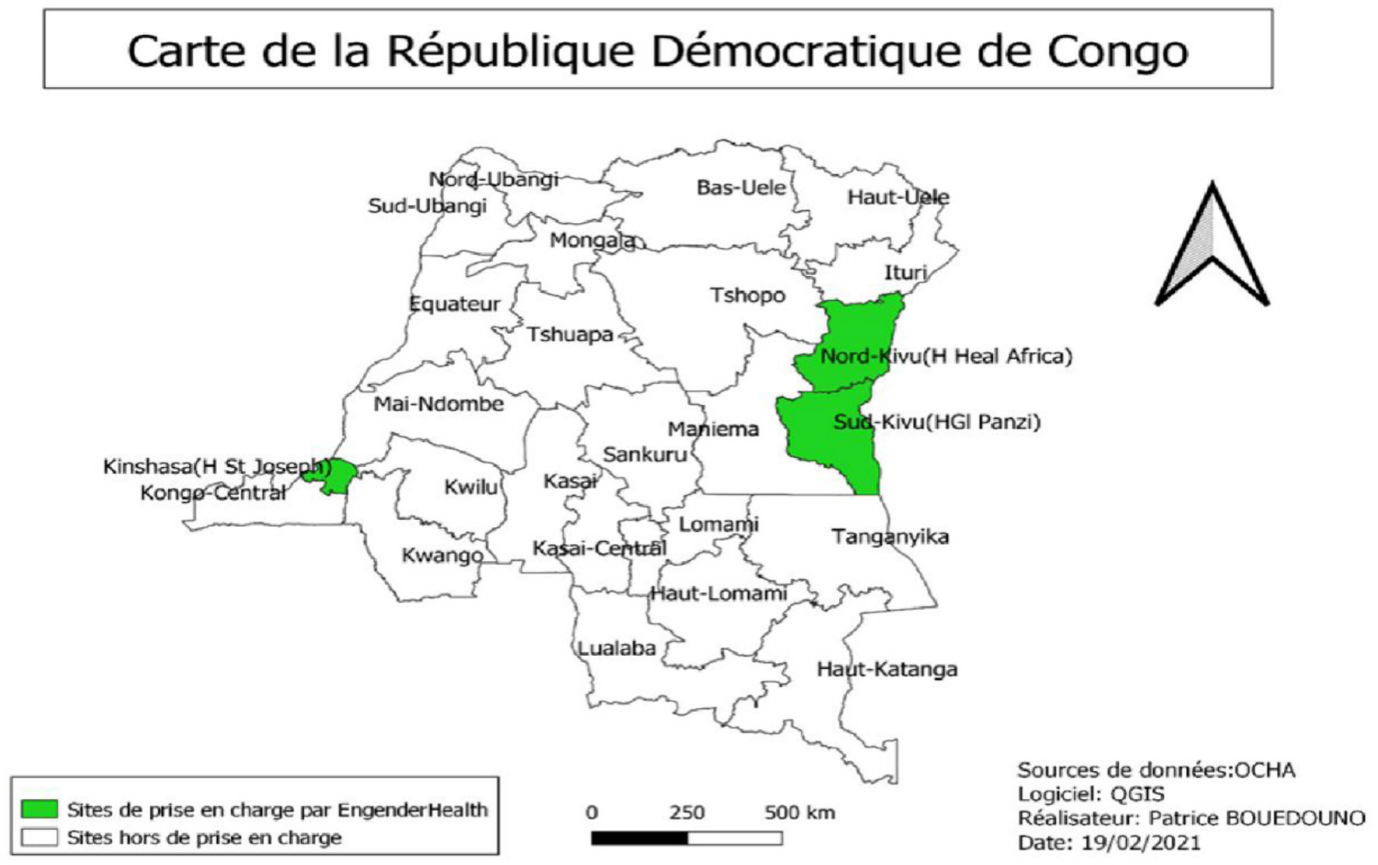
Map of the DRC with study sites.

Data collection took place in Kinshasa (EngenderHealth's office and the St. Joseph's Hospital care site) and at other care sites in the countryside (Panzi General Hospital in Bukavu in South Kivu Province, and Heal Africa Hospital in Goma in North Kivu Province) (Figure 1). These care sites have respective capacities of 300 beds, 450 beds, and 220 beds.

### Quantitative Methods

#### Population and Study Period

The quantitative data collection focused on women treated for FGF between January 1, 2017 and December 31, 2019 at all sites of the study. It consisted of evaluating the presence, completeness and accuracy of the filling of the CT forms.

#### Sampling

Systematic random sampling was used to select the CT of women undergoing FGF care. For feasibility, 75 records (25 in each site) were selected from a sampling frame of 923 charts. Each patient record was to contain seven forms that comprise the CT (Table 1).

**Table 1:** Forms included in the Client Tracker.

**N**.	**Name of the record**	**Description**	**Senior manager**
1	Eligibility form for surgery on admission	Patient's identity, history, physical examination and para-clinical examinations.	Medical doctor
2	Surgical candidacy form	Vital signs, history, results of para-clinical examinations	Anesthetist and surgeon
3	WHO Surgical Safety checklist	Checklist of procedures performed before anesthesia, before skin incision and before leaving the operating room	Anesthetist and operating room nurse
4	Operating protocol sheet	Describes the fistula and the operative acts and procedures	Surgeon
5	Patient transport record	Checklist for transport from the hospital ward to the operating room, from there to the postoperative recovery room, and from there to the hospital ward	Nurse: ward and operating room
6	Daily postoperative care sheet	Vital signs, 3 Ds (Drink,Drain et Dry), other symptoms, medications	Ward nurse
7	Discharge summary form	Patient identity, summary of admission, sentinel events, medications at discharge, contraceptive method chosen	Ward nurse

#### Description of the Client Tracker Forms Used in the FC+ Sites

The CT comprises seven forms, filled out by various staff in different departments involved in FGF management (Table 1). The CT is designed in hard (paper) and electronic versions. The different clinicians in the respective hospital units fill in the hard version of the CT forms, serving as a source document for the collected information. The information on the hard version of the CT is then entered by encoders (not necessarily clinicians) into the electronic version.

#### Data Collection

Data collection entailed checking the presence of each CT form (paper version) in the patient's record and the completeness of each information reported in the form. A CT form was considered to be present when the research team found it in the patient's record. A form was deemed to be complete if it contained all the information requested on the form. A form was correctly completed if all the information it had was free of mistakes and readable (no erasures or overwriting).

The data were collected in February 2020 using a predefined Microsoft Excel file.

#### Data Management and Analysis

Data were analyzed using Microsoft Excel Windows 16 to present descriptive findings on the proportions of forms present, completely and correctly filled out.

### Qualitative Methods

#### Participants and Recruitment

Participants in the qualitative study were recruited purposively, to reflect variations in their functions in the FC+ project. They were recruited at the three FC+ care sites and the EngenderHealth office. Participants from the FC+ sites included Ward Nurses, Surgeons, Data Managers, and Project Managers. At the office, the Senior Program Manager, Technical Advisor and a Data Manager were interviewed. Interviews were arranged at times and places that were convenient for participants.

#### Data Collection

Data were collected in January 2021 through individual in-depth interviews using an interview guide. Interviews were held either face-to-face or virtually. The interviews were conducted by one of the study co-authors (BSC), who is a social and health research scientist. Each interview was recorded following informed consent from each participant.

The following aspects were explored during the interviews: participant's familiarity with the CT, training on the use of the CT, integration of the CT in FGF management, changes attributed to the use of the CT in collection of quality data and FGF management, opportunities for and challenges to CT sustainability in FGF management.

#### Data Management and Analysis

Interviews were fully transcribed into French and analyzed using deductive and inductive coding. Three coders coded the transcripts concomitantly, with three debriefing sessions (before, during and at the end of coding) that helped develop a consistent interpretation of the codes among the analysis team. Deductive codes (based on existing literature) were first applied to the data. Next, inductive codes were detected from new themes emerging from interviews with informants. The codes were classified into main categories and sub-categories based on differences and similarities according to participants' characteristics. Data were analyzed using Windows Word 16, Excel 16.

### Ethical Considerations

Informed consent was obtained from each participant prior to each interview. In addition, the study protocol was approved by the Health Research Ethics Committee of the DRC (ref: Approval Number ESP/CE/153/2020).

## Results

### Participants' Characteristics

Overall, 14 key informants participated in the qualitative study (Table 2). Heal Africa (*n* = 5) and Panzi (*n* = 4) were the most represented care sites. Data Managers (*n* = 4) and Surgeons (*n* = 4) were the most frequently represented cadre of participant. These participants were majority female (*n* = 8).

**Table 2:** Characteristics of participants in the qualitative study (*N* = 14).

**Characteristics**	**Number**
**Structure**	
EngenderHealth Office	3
Heal Africa Site	5
Panzi Site	4
Saint Joseph Site	2
**Function**	
Senior Project Manager	1
Technical Advisor	1
Project Manager	3
Data Manager	4
Surgeon	4
Nurse	1
**Sex**	
Female	8
Male	6

### Integration of the Client Tracker in FC+ Sites

CT integration in FGF management at FC+ sites was primarily done through staff training on CT use and gradual adaptation of the CT forms to the care context.

#### Training on Client Tracker Use

One of the key elements of CT integration into FGF management at the FC+ sites has been staff training on CT use. The staff involved in FGF management at the three sites, regardless of their roles in the project, were trained during workshops organized at each sites. The staff included health workers (surgeons, nurses, anesthesiologists) and administrative personnel (project managers and computer specialists). National experts from the EngenderHealth office facilitated the training.

“*Yes, I was trained on Client Tracker by the EngenderHealth's national team, which came from Kinshasa.”* Nurse, Heal Africa

However, a few staff reported that they did not participate in this training workshops as they were either unable to attend or joined the care teams/project after the workshops were organized. Nevertheless, some of these staff reported that they have progressively learned how to use the CT through personal (self-training) practical exercise during FGF management.

“*You have to do it on your own… By using it once, twice or more, we've understood and we're able to progress…”* Data Manager, Saint Joseph

Teamwork and supervision from EngenderHealth's office team also contributed to the learning of the project staff on CT use.

“*They came to see how things were going to address the difficulties we were facing with the use of the tool.”* Data Manager, Saint Joseph

Focused training was planned for Data Managers in March 2020; however, this was postponed because of the Covid-19 pandemic.

“*We [planned] a training for all the data clerks, but last year, with the Covid-19 advent, it turned things upside down.”* Technical Advisor, EngenderHealth Office

Respondents recommended training sessions for staff who had not benefited from the original training with experts. Some respondents also requested additional training on fistula counseling, as existing counseling training focuses on family planning.

#### Progressive Adaptation of the Client Tracker

The CT was also integrated into FGF management through progressive adaptation of the CT forms accounting for realities in the different management units, and additional or refined translation into French of some sections on the CT forms.

“*We have changed the sections into French and have adapted them accounting for the unit… there were other elements that we should add… we had to practice a lot more to be able to correct the mistakes.”* Data Manager, EngenderHealth Office

### Client Tracker Use

**Figure 2:**
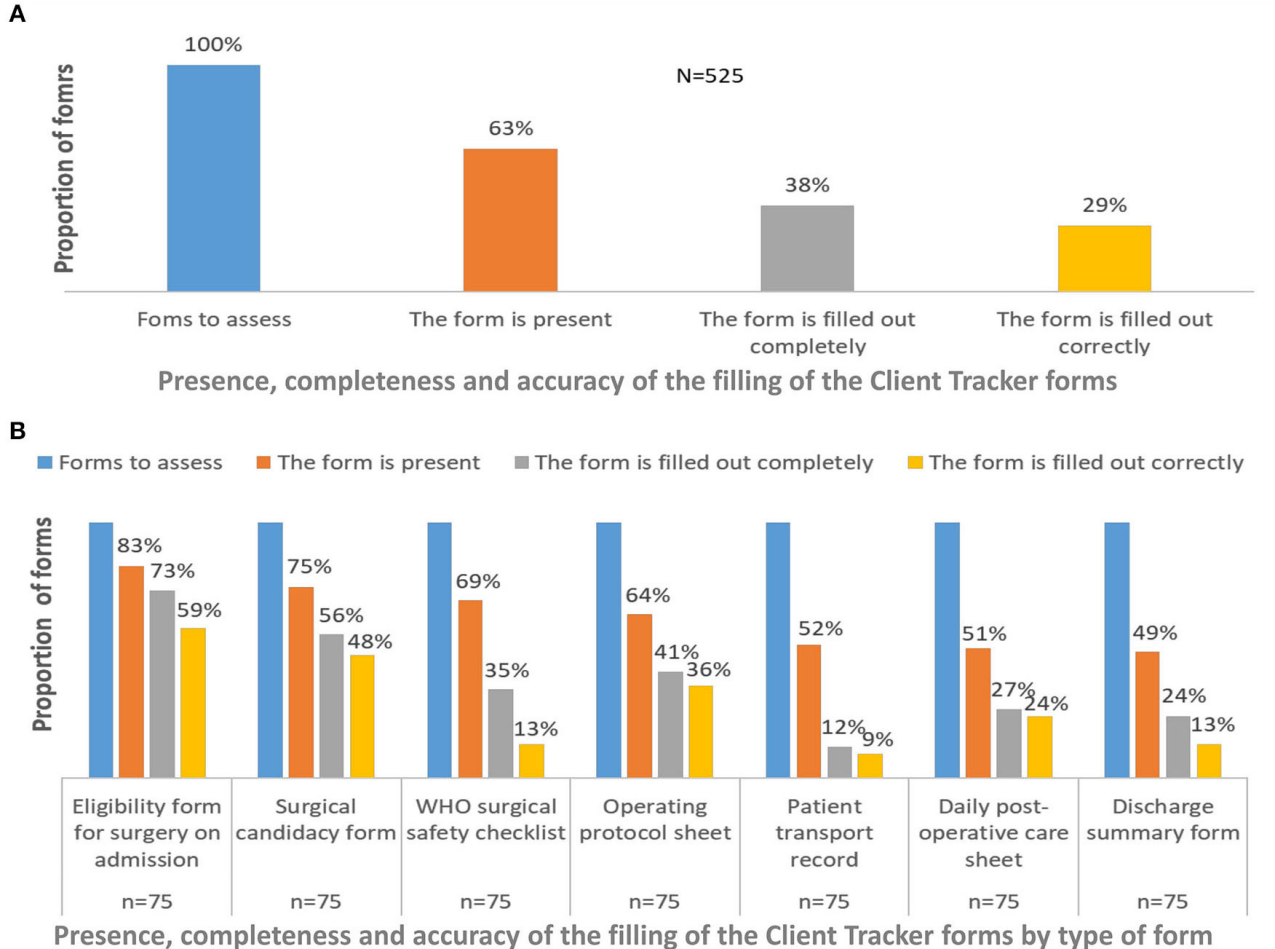
Client Tracker use by type of form in female genital fistula management at Fistula Care Plus Sites, DR Congo, 2017–2019.

The quantitative analysis assessed the use of CT at FC+ sites (Figure 2). Of a total sample of 525 expected forms (seven forms per record, as described in Table 1), 63% were present in patients' medical records, 38% were completely filled out, and 29% were filled out correctly (A). In terms of assessment per type of form, the eligibility form for surgery on admission was the most present at the sites (83%), followed by the surgical candidacy form (75%), WHO surgical safety checklist (69%), and operating protocol sheet (64%) (B).

The proportion of forms present decreases throughout the patient's pathway, from 83% at admission to 49% at discharge. The completeness of the third through seventh was below the cross-form average, between 12 and 41%. The proportion of correctly completed forms drops significantly below the cross-form average from the third form onwards (13–25%).

In general, forms completed after surgery and in the peri-hospital discharge by nurses showed the lowest proportions of presence, completeness, and accuracy.

### Perceived Contributors to Client Tracker Use

Four main themes identified through qualitative analysis provide a better understanding of CT use by the staff. These themes include familiarity with CT, disparity in understanding concepts used in the CT forms, excess workload related to the CT, and overload of the CT forms.

#### Familiarity With the Client Tracker

Respondents said they are familiar with the CT. For health workers, their familiarity with the CT is related to the fact that they have been using it for FGF management for 3 years (since 2017).

“*I handle the Client Tracker almost during every [surgical] procedure.”* Surgeon, Saint Joseph

Among EngenderHealth office staff, their level of familiarity was associated with their participation in CT integration into the sites.

#### Disparity in Understanding and Points of Confusion

Respondents noted that staff with clinical training fill out the CT forms more quickly than other staff, such as computer specialists. Indeed, the terminologies used in these forms are mostly biomedical and clinical.

“*Our site has chosen a data clerk who is a doctor… who understands the terminology… but for other sites, they are computer specialists.”* Data Officer, Saint Joseph

Another challenge that has hindered the CT's optimal use includes confusion caused by the presence of sections for conditions other than fistula. For example, EngenderHealth office noted that the CT includes prolapse and incontinence, which was felt to confuse users.

“*We need to continue to adapt the forms… We have suggested some modifications to the Client Tracker… the Client Tracker mixes up fistulas, prolapse and incontinence; sometimes the information gets confused.”* Data Manager, EngenderHealth Office

Additionally, when the CT was newly introduced, some sections were poorly understood by the staff because they were in English.

#### Additional Workload

Respondents reported that a challenge in using the CT is the large number of forms and sections. Therefore, filling out all these forms creates an additional workload for care site staff, especially clinicians, such as surgeons or nurses. This contributes to a lack of completeness of forms.

“*The forms were many, and it was difficult to complete all this at the same time.”* Nurse, Heal Africa

Respondents suggested that there should be a staffperson dedicated to ensure the filling of the CT forms.

“*For the information to be recorded in real-time to avoid missing data, it is recommended to have a person dedicated to this task”*. Senior Program Manager, EngenderHealth Office

### Contribution of the Client Tracker to Fistula Care

According to respondents, the CT has mainly contributed to improving data and reporting between the different care sites and the EngenderHealth office, quality of care, follow-up of fistula patients, and self-supervision of care activities by providers.

#### Improvement of Data Quality and Reporting

In terms of improving data quality, the CT has contributed to the standardization and organization of patient data, facilitating the cross-checking among the three sites. It has also contributed to reducing missing data for variables useful for research.

“* Client Tracker has helped us to improve data quality because data needs to be verifiable… Client Tracker works well in the sites, in the sense that we have data that we can present in a more orderly fashion.”* Technical Advisor, EngenderHealth Office

In terms of data reporting, the CT made it possible to send the data more quickly to Project Managers and to the EngenderHealth office.

“*This allowed us to have the data as soon as possible, compared to what was before.”* Senior Program Manager, EngenderHealth Office

Monthly data reporting, facilitated by the CT, has helped strengthen communication between staff in the different sites and the EngenderHealth office.

“*This monthly report has created a more frequent communication within the staff and between the site and the central team in Kinshasa*. Project Manager, Heal Africa

#### Improvement of Care Quality

Respondents also reported that the CT contributes to improving the quality of care because it serves as a checklist for staff to ask all necessary questions to the patient. It also contains sections that allow for collecting specific additional information on the diagnosis of fistula.

”*…with the Client Tracker you get the chance to ask all the questions.“* Nurse, HEAL Africa

”*We have a protocol that is not complete about the diagnosis, but the specificity of the diagnosis can be found in the Client Tracker.“* Project Manager, Heal Africa

#### Improvement of Patients Follow-Up

Respondents noted that the CT's contribution to improving patient follow-up lies in delivering comprehensive data on the patient from admission to discharge and even after discharge from the treatment center.

”*It [the Client Tracker] is a database that can track the patient's entire pathway at hospital and gives us all the necessary information from the patient's admission to discharge and even afterwards, and it even describes family planning.“* Nurse, Heal Africa

”*It's because we have individual data that allows us to understand the different cases of each woman who has been operated on for a second or third time.“* Technical Advisor, EngenderHealth Office

#### Self-Supervision

The CT favors self-supervision among staff at each care site, as it raises their awareness to double check the various tasks they perform daily, discuss them as a team, and thus continuously improve them.

”*… It also allows us to be aware of how we fulfill the various tasks in the service.“* Data Officer, Saint Joseph

”*It's the fact that they hold meetings and decide to make decisions unlike before the introduction of Client Tracker.“* Senior Program Manager, EngenderHealth Office

### Client Tracker Sustainability

Qualitative findings regarding CT sustainability include both opportunities and recommendations.

According to respondents, the foremost opportunity for CT sustainability is that the CT is perceived as useful by hospitals managers where FGF management is integrated.

”*As our leaders have approved, this will continue because it will improve the quality of our data.“* Nurse, HEAL Africa

The main recommendations made by respondents for CT sustainability included the involvement of the Ministry of Health in scale-up and integration, and continuous external financial support.

”*The Ministry of Health has to be a collaborator who will help out make the Client Tracker work and make [it] a document that could be, perhaps, adopted in different sites“*. Data Manager, Saint Joseph

Some respondents found that integrating the CT into FGF management is costly; hence, they suggested financial support and availability of material and human resources for sustainability.

”*Using the Client Tracker is costly, so we [need] additional means for sustainability, because the papers [we need] are costly"*. Senior Program Manager, EngenderHealth Office

## Discussion

The present study is one of the first to document the experience of integrating a data collection tool into the routine management of conditions that require surgical care in sub-Saharan Africa. It suggests that the CT has been integrated into the routine management of FGF in DRC. It also shows that the CT has the potential to improve data quality and reporting, patient care, patient follow up, self-supervision, and communication among health care workers. However, CT integration in FGF management induces an additional workload that is difficult for some staff to manage, leading to limitations in the completeness and correct filling of the forms. In addition, staff training on CT use differs from a team to another. These results have implications in terms of policies and practices for improving FGF management in the DRC and possibly in other countries with similar contexts.

This study suggests that the CT is a tool that can be integrated into and improve routine FGF management. Three main factors can explain the contribution of CT to improving FGF management at FC+ sites. Firstly, thanks to the comprehensiveness of the CT forms as reported by the study respondents, health care workers can conduct a more informative and exhaustive client assesment, which may lead to better clinical practices and medical follow up. This implies that the use of CT leads to an improvement in the quality of care in the different hospitals that use it. Second, the different CT forms are filled out in different units at each site. The collection of comprehensive information in all these units throughout the patient's hospital pathway is crucial to improving patient follow-up from admission to discharge and even in the postoperative period. Third, the data collected through the CT are more organized, standardized across care sites, and forwarded in a timely manner to the EngenderHealth office. This supports joint and regular supervision of care activities at different sites, which can more effectively inform decision making to improve fistula care, as clinical data quality has been reported as key to improving health care management (14, 15).

The study also showed that the CT can be sustained for FGF management at FC+ sites in DRC. The potential for CT sustainability lies in the perceived usefulness of the tool by users and the fact that it is already integrated into the routine management activities. However, several conditions must be met to ensure this sustainability. First, the MoH must be fully involved in the scale-up and use of this tool at the national level. The State's adoption of a health intervention would better guarantee its sustainability; however, this guarantee may be mainly based on financial resources (16–18). In addition, to ensure CT sustainability for FGF management, it is crucial to address the workload related challenge. Respondents said that the CT induces additional workload, which is sometimes difficult to manage. Excessive workload among health workers has been documented in different contexts as affecting the quality of health services (19–21). A review of the CT forms involving staff at operational and programmatic levels can support shortening of the forms and their harmonization with existing routine forms. This would reduce the time required to fill out the CT and the number of forms to be filled out for FGF management.

This study also reveals that some staff in charge of filling out the CT forms mastered them better than others. The lack of mastery of filling techniques by some staff could lead to errors in the information reported in the CT forms.—In the discussion, it would be useful to discuss the accuracy of the information being collected in the forms. This is especially needed as it is reported that not all those using the forms have received formal training and have had to train themselves. The lack of mastery of filling techniques can also slow down the completion process. Assessment of CT use showed a decrease in the presence and completeness of the forms and the quality of the reporting along the care pathway, from the first to the seventh form. Variations in the quality of the reporting on the forms were related to the forms' characteristics (length, type of information to collect), the staff (profile, workload) or the unit (staff, tasks, and environment) throughout the patient's care pathway from admission to discharge. Therefore, it is necessary to establish a continuous training program for the staff in charge of filling out the CT, accounting for the different profiles of the staff. Continuous staff training is known to be essential for maintaining health services quality (22–24).

This study has limitations. First, the quantitative analysis did not include data on the pre-CT period at the sites to measure the CT's effect on data quality and FGF management. Second, quantitative data on the use of the CT was collected retrospectively; this approach did not allow us to check accuracy of the information reported in the CT. Third, the qualitative data were limited to staff supported by the FC+ project and it is possible that social desirability biases (25) influenced the study findings. Nevertheless, this study has several strengths. First, it combined two complementary quantitative and qualitative methods, allowing a better understanding of the research question and reciprocal compensation of methodological limitations. Second, this study followed the COREQ (Consolidated Criteria for Reporting Qualitative Research) guidelines (26) for the qualitative component. Third, this study was designed through a participatory operational research initiative conducted by the FC+ project, and has enabled the generation of practical recommendations that are appropriate to guide FGF management programs in DRC.

## Conclusion

This study shows that the CT can be integrated into the routine management of FGF in DRC and indicates that the CT can improve data quality and reporting, patient care, patient follow-up, self-supervision, and communication among the staffs. However, FGF integration into FGF management induces an additional workload that is difficult for some staff to handle, and staff level of training on and comfort with CT use differs within and among care teams.

The CT provides an appropriate database for continuous evaluation and scientific research; this could help guide the country's efforts for FGF management. Nevertheless, a simplification of the CT and or harmonization of its content with existing routine forms, coupled with adequate in-service training of staff in charge to fill out the forms, would further maximize CT effectiveness and sustainability.

## Data Availability Statement

The raw data supporting the conclusions of this article will be made available by the authors, without undue reservation.

## Ethics Statement

The studies involving human participants were reviewed and approved by the Health Research Ethics Committee of the DRC. Written informed consent for participation was not required for this study in accordance with the national legislation and the institutional requirements.

## Author Contributions

The study protocol was developed by JP, EK, and CF and reviewed by VT, BC, and AD. Data collection was ensured by JP, EK, CF, BC, RB, and PH. BC did the data analysis. The first draft of the manuscript was written by JP, EK, CF, RB, PH, and DB and critically reviewed by BC, SS, VT, and AD. All authors were involved with interpretation and read and agreed to the final version of this manuscript.

## Funding

The study and related manuscript development were funded by the United States Agency for International Development (USAID) under Associate Cooperative Agreements AID-OAA-A14-00013 and 7200AA20CA00011.

## Author Disclaimer

The opinions expressed are those of the authors and do not necessarily reflect the views of USAID, or the United States Government.

## Conflict of Interest

DB and VT were employed by EngenderHealth. The remaining authors declare that the research was conducted in the absence of any commercial or financial relationships that could be construed as a potential conflict of interest.

## Publisher's Note

All claims expressed in this article are solely those of the authors and do not necessarily represent those of their affiliated organizations, or those of the publisher, the editors and the reviewers. Any product that may be evaluated in this article, or claim that may be made by its manufacturer, is not guaranteed or endorsed by the publisher.
